# Evaluation for the operational risk factors of public transportation process based on entropy weighted- DEMATEL method

**DOI:** 10.1371/journal.pone.0323417

**Published:** 2025-05-16

**Authors:** Xiaoyan Qi, Xin Huang, Wenlong Zhu

**Affiliations:** 1 Nursing School, Anhui Medical University, Hefei, Anhui, China; 2 Management School, Anhui University, Hefei, Anhui, China; Sichuan University, CHINA

## Abstract

Identifying and assessing the key factors that influence the operations of public transportation from an operational risk perspective is a critical challenge. In this paper, in order to tackle this issue by dissecting 18 risk factors through a comprehensive lens that encompasses five dimensions of the public transportation operation: infrastructure completeness, operational procedures, management practices, process requirements, and risk mitigation strategies, each with its respective evaluative metrics. To offer a nuanced analysis of these risk factors, we introduce an entropy weight-based DEMATEL (Decision-Making Trial and Evaluation Laboratory) method. This approach leverages the entropy weight decision-making process to quantify the significance of each risk factor, acknowledging the inherent uncertainties in the evaluation process. By applying the DEMATEL method to the collected data, it becomes evident that safety consciousness stands out as a pivotal factor, with human initiative emerging as a central element in the operational flow of public transportation. Furthermore, our study reveals that substantial funding, technological advancements, regular inspections, and stringent regulations are critical risk factors that warrant considerable attention in the management of public transportation operations. The findings underscore the necessity for a multifaceted approach to risk management to bolster the safety and efficiency of public transportation services. To foster a safer and more effective public transportation system, it is imperative for stakeholders to not only heighten their vigilance regarding safety but also to ensure that adequate financial resources and regulatory frameworks are in place. Only through a holistic and multidimensional risk management strategy can we hope to minimize the incidence of public safety risks.

## 1. Introduction

The rapid economic growth has resulted in increased frequency and ease of interpersonal communication [1]. The introduction of various virtual networking technologies has yielded significant time and resource savings, yet certain activities necessitate direct, face-to-face interaction. These interactions cultivate robust trust among parties and foster collaborative endeavors [2]. Advancements in transportation infrastructure have diversified travel options for individuals. Notably, China’s considerable investments in infrastructure development have garnered international acclaim and yielded numerous contracts for high-quality roads and bridges. However, the accelerated pace of urbanization has generated diverse travel needs [3], including age-specific public transportation and subway requirements. A sudden surge in short-term travel demand can strain transportation capacity, posing significant challenges to public transportation systems. Therefore, effectively addressing and reconciling emerging conflicts in public transportation demand is crucial.

Operational challenges in public transportation stem primarily from the complex and fluctuating nature of travel demand [4]. Travelers’ journeys are becoming increasingly complex due to continuous improvements in quality of life, often outpacing research and development efforts of service providers in delivering high-quality travel experiences. This demand-supply mismatch perpetuates recurring issues in travel services. However, analyzing and predicting traveler behavior has alleviated issues such as traffic congestion and insufficient public transport capacity during peak hours, enhancing green travel services [5]. Resolving these cyclical contradictions is crucial. Identifying practical strategies within public transportation’s operational framework has proven effective. Effective strategies should be implementable and manageable. From a transportation operation safety perspective, public transportation’s safety impact is broader and more significant than that of individual transportation vehicles [6]. Strategically examining operational risks in public transportation yields effective measures. This approach streamlines operations, ensuring manageability.

Public transportation systems rely heavily on robust infrastructure for effective operation [7]. The absence of high-quality public facilities poses significant safety risks [8]. Moreover, the expertise of personnel involved is crucial, as their proficiency underpins public transportation safety. Professional training and adherence to compliance standards substantially mitigate operational risks during service delivery. Establishing comprehensive rules and regulations is essential for managing public transportation, ensuring the safety of systems, passengers, and cargo. These guidelines must be mandatory for all sector stakeholders, reducing operational risks from an institutional perspective. Supportive policies from management departments can sustain service provider motivation. Implementing reward and penalty systems fosters standardized practices among enterprises. From a policy and institutional standpoint, regulatory legitimacy and enforcement are critical in preventing risk [8]. For operational entities in the public transportation sector, maintaining a strong reputation is vital for delivering quality services, ensuring a steady passenger and freight base. Companies with sound operational and financial health are better equipped to withstand external financial uncertainties. External factors, such as extreme weather events and geopolitical issues (e.g., sudden energy price hikes due to conflicts in oil-producing nations), can significantly impact public transportation services, affecting operational costs. Minimizing the impact of unforeseen external risks requires a proactive approach, characterized by a sense of crisis and prompt action. Furthermore, the quality of vehicles and equipment in public transportation significantly influences the overall travel experience. Effective quality risk management is crucial, as it profoundly affects the ongoing operations and success of involved enterprises. Managing public transportation’s risk-prone operational process necessitates mitigating the cumulative effects of various uncertainties. A thorough analysis of operational risk factors from an operational standpoint is vital. To provide a robust decision-making reference basis, assessing operational risk factors in public transportation is essential. To better understand operational risk factors affecting public transportation, two key questions must be addressed: (1) How can comprehensive operational risk factors be identified? and (2) What method can provide a scientific foundation for analyzing the selected risk factors?

To address the two questions raised in this paper, the research conducts an analysis of the background involved in the impact factors of the public transportation operation process, establishing a realistic foundation for subsequent impact factor screening. Additionally, this study proposes a combined research method, integrating the entropy weight method and the DEMATEL decision-making method, for analyzing risk impact factors. This approach leverages two key advantages: the objective weight analysis provided by entropy weight and the causal relationship analysis offered by DEMATEL in system factor analysis. Combining these methods provides a more scientific basis for decision-making (The entropy weight-DEMATEL analysis process, through in-depth parsing of the importance and causal relationships of public transportation operation factors, can deeply explore the true role of the influencing factors.).

This paper presents a comprehensive analysis of factors influencing public transportation’s operational process, identifying primary risk factors significantly impacting system functioning. The study evaluates the degree of influence of these factors and explores their interrelationships. To achieve a robust assessment, the entropy-weighted DEMATEL (Decision Making Trial and Evaluation Laboratory) combination decision-making method is employed. This approach integrates quantitative and qualitative aspects, providing nuanced insights into complex dynamics. By applying this method, the study aims to provide public transportation decision-makers with actionable insights and an evidence-based decision-making framework. This can inform effective risk management and operational optimization strategies, ultimately enhancing public transportation service safety, efficiency, and sustainability. The structure of this paper unfolds in a systematic progression, delineated as follows: The second section provides a comprehensive literature review, synthesizing current scholarly insights. The third section subsequently delves into an analytical examination of the risk factors inherent in the operation of public transportation systems. The fourth section elucidates the entropy-weighted decision-making trial and evaluation laboratory (DEMATEL) method, which integrates it with a composite analysis approach. The fifth section presents an evaluative framework for assessing risk factors within the context of public transportation safety operations. In conclusion, the sixth section encapsulates the findings and offers implications for future research and practical application.

## 2. Literature review

Although the current body of research does not offer a direct precedent for this study, there exists a corpus of literature that intersects with our topic from the vantage points of transportation and risk factors. This literature provides a foundational context that is essential for our inquiry. Furthermore, the methodologies of entropy weight analysis and the DEMATEL have demonstrated their utility in related fields, which makes them pertinent to our research. Their insights are anticipated to enrich our analytical framework and contribute to a deeper understanding of the subject matter. Consequently, the literature review incorporates the following relevant works.

### 2.1 Transportation-related research

Transportation plays a vital role in daily life, providing diverse travel options essential for individual mobility and societal functioning. As a well-established field of study, transportation has been extensively examined from various scholarly perspectives, each contributing uniquely to the discipline’s advancement. The selection of transportation modes for different goods depends on factors such as the nature of the goods and logistics of transportation routes. For example, railway transportation is often preferred for fragile items like glass [9]. Establishing robust traffic evaluation criteria is crucial for ensuring traffic system resilience under significant congestion. The strategic positioning of key nodes in transportation networks is influenced by geographical considerations and the reliability of interconnections. In energy transportation, identifying more efficient, low-carbon pathways is of paramount importance [10]. Accurate energy estimation is critical, governing consumption and guiding transportation development strategies [11]. When constructing new transportation infrastructure, such as bridges, investment risks and safety hazards posed by environmental unpredictability must be considered, including costs associated with overcoming transportation challenges and facilitating societal recovery after extreme weather events [12]. In public emergencies, establishing a multi-modal, radiative transportation network offers an efficient and cost-effective solution, enhancing emergency management. Identifying key transportation hubs within multi-modal networks is instrumental in optimizing overall network performance [13]. Although numerous studies exist in the transportation sector, most focus on transportation modes, efficiency enhancement, and energy usage. However, a notable research gap exists regarding public transportation, particularly in understanding the operational dynamics of the entire transportation process, presenting an opportunity for exploration in this study.

### 2.2 Risk factor research

The literature on risk factors is extensive, stemming from uncertainties in internal and external environments. Numerous studies have investigated risk factors from diverse perspectives. Due to space limitations, this paper highlights a selection of recent studies pertinent to risk factors. Investment and development in renewable energy sectors involve inherent risks; thorough evaluation of these factors can mitigate risks and minimize losses associated with investment uncertainties [14]. Quantitative analysis of groundwater pollution sources, coupled with health risk monitoring and assessment, accurately determines health risks posed by these sources [15]. Assessing human operational risks associated with medical devices enhances their operational efficiency [16]. Evaluating risk factors related to sustainable innovation technologies in green buildings ensures their long-term, stable development [17]. Effective communication of landslide risks and underlying causes is crucial for early warnings on geological hazards and preventing unnecessary losses [18]. Notably, while existing literature on risk factors focuses on model construction and empirical research for measurement and assessment, comprehensive analyses on operational dynamics of the subjects under study are relatively scarce. This knowledge gap presents an opportunity for this paper to conduct a holistic analysis of the public transportation operation process.

### 2.3 Applications of entropy weight analysis and DEMATEL research

#### 2.3.1 Applications of entropy weight analysis.

The entropy weight analysis method is widely employed in evaluation and decision-making contexts due to its effectiveness. Its primary advantage lies in its capacity to objectively quantify evaluation criteria weights. By determining these weights, the relative significance of each criterion is established, providing valuable insights for analytical decision-making. The method’s popularity stems from its ability to objectively determine evaluation indicator weights. In the context of material extraction from recycled ceramics in coal lime processes, integrating the entropy weight method with the Technique for Order Preference by Similarity to Ideal Solution (TOPSIS) effectively mitigates subjective bias in indicator weighting [19]. The entropy weighted method serves as the foundational step for TOPSIS analysis, calculating weights [20]. The entropy weight analysis method is particularly beneficial in performance evaluation, as it considers criteria weight values [21]. Combining entropy weight and fuzzy TOPSIS effectively assigns weights to criteria and ranks corresponding strategies [22]. Based on the preceding discussions, the entropy weight method is proposed for evaluating public transportation operation risk factors. This approach ensures a balanced and data-driven assessment, enhancing decision-making process reliability.

#### 2.3.2 Applications of DEMATEL research.

The Decision Making Trial and Evaluation Laboratory (DEMATEL) method is a valuable decision-making tool, particularly for analyzing causal relationships among decision elements. Its analytical framework primarily compares the degree of pairwise influence among factors. The DEMATEL method has diverse applications, with its utility demonstrated in the domain of medical accidents through quantitative analysis of causal relationships in human-induced incidents [23]. Moreover, DEMATEL assesses not only the causal dynamics of evaluation criteria but also their relative weights to a significant extent [24]. By quantifying causal relationships between factors, DEMATEL provides a solid foundation for understanding their interaction mechanisms [25–29]. This strength of DEMATEL in causal analysis provides a robust framework for uncovering intrinsic connections between risk factors impacting public transportation operations. The method elucidates the complex web of causality, facilitating identification of primary factors and comprehension of their interrelationships and reciprocal influences. This comprehensive approach significantly enhances risk management and decision-making efficacy in public transportation systems by addressing root causes of issues.

In summary, although scholars related to traffic operation, influencing factors, and entropy weight and DEMATEL have done relevant research work, at present, the analysis of influencing factors of traffic operation is relatively less, and more is analyzed from a relatively short-term perspective, with relatively fewer research references proposed. At the same time, the combination of entropy weight and DEMATEL research has certain advantages in the analysis of the importance and causal relationship of influencing factors, which provides the possibility for utilize for corresponding analysis in the combined evaluation research, hence, in this paper, we can combine the advantages of entropy analysis in subject weight and DEMATEL in causal relations analysis to compose the entropy weight-DEMATEL method for comprehensive influencing factor analysis from the perspective of public transportation operation.

## 3. Operational risk factors that affect public transportation

From the perspective of public transportation operations, this paper seeks to identify and select key risk factors that substantially impact the operational process. The objective is to perform a comprehensive analysis acknowledging the multifaceted nature of these risks, thereby ensuring assessment results are both accurate and practically applicable to real-world scenarios.

Effective operation of public transportation systems relies on the presence of comprehensive infrastructure, a fundamental prerequisite that necessitates early and strategic planning [30]. This preliminary planning establishes the foundation for efficient transportation services. The quality of transportation infrastructure serves as a critical metric for evaluating regional mobility capabilities and contributes significantly to overall development. Concurrently, cultivating specialized expertise within this sector is equally crucial, particularly the development of research and development (R&D) and design professionals. Their expertise is indispensable for ensuring sustainable growth in transportation projects. Stringent material selection regulations must be implemented during infrastructure construction to prevent potential quality and safety issues associated with substandard components. Construction processes should balance practical considerations with environmental concerns, prioritizing eco-friendly materials to minimize pollution [31]. Before infrastructure deployment, rigorous testing is essential to verify functionality and safety [32]. Furthermore, regulatory bodies’ approval processes can facilitate infrastructure eligibility for policy incentives, thereby promoting development. In summary, the establishment of transportation facilities enhances regional accessibility and provides a solid foundation for public transportation systems operation.

Standardized personnel conduct is crucial for ensuring the safety of individuals and property within the public transportation sector. Achieving this requires a multifaceted approach centered on enhanced construction quality, proficient operational skills, and a steadfast commitment to safety [33]. To optimize personnel operations, the development of comprehensive operational guidelines, detailed process manuals, and explicit job requirements is essential. Effective supervision and management protocols, supplemented by a well-defined system of incentives and penalties, are also vital for maintaining elevated standards. Regular training sessions, conducted by relevant departments and enterprises, foster proficient operational skills among personnel. These sessions focus on updating operators on the latest operational requirements and familiarizing them with necessary procedures and policy measures. However, safety awareness remains the paramount consideration in personnel operations. In the absence of a robust safety culture, the risk of traffic accidents during operational processes increases substantially. Therefore, prioritizing safety awareness training and cultivating a pervasive safety culture across all aspects of public transportation operations is imperative.

From the perspective of traffic operation management departments, the implementation of well-defined traffic rules and regulations is crucial for efficient traffic operations [34]. These rules provide a standardized framework for operational practices within organizations. Firstly, comprehensive traffic regulations establish a detailed structure guiding operational standardization for transport companies, outlining protocols and procedures that ensure compliance and professionalism [35]. Secondly, traffic rules and regulations play a key role in shaping behavioral norms by setting clear expectations for conduct, thereby promoting safety and efficiency among all road users, including drivers, passengers, and pedestrians [36]. Furthermore, rules and regulations serve as a guiding tool for individuals and organizations [37], providing clarity on permissible actions and responsibilities, facilitating navigation of traffic management complexities, and enabling anticipation and mitigation of potential risks. In summary, effective traffic rules and regulations form the foundation of a well-functioning traffic system, ensuring order, safety, and predictability for all stakeholders.

The effectiveness of public transportation operations relies on the collaborative efforts of individuals utilizing various transport vehicles to serve the public [38]. Public transportation services are typically delivered by contracted enterprises. These contracted enterprises may be eligible for transportation policy incentives, which can stimulate enhanced service delivery and operational efficiency. From the perspective of transportation operation management departments, the implementation of effective supervision and management strategies is essential. Such oversight plays a critical role in ensuring the safety and quality of transportation operations, guaranteeing that contracted enterprises adhere to rigorous service delivery standards. This fosters a reliable and secure transportation environment for the public. In essence, a harmonious relationship between contracted enterprises and transportation operation management departments is vital for successful public transportation operations. Effective oversight and support facilitate collaborative efforts, enabling these entities to provide safe, efficient, and customer-centered transportation services.

The selection of qualified and reputable enterprises is vital for the efficient operation of public transportation systems. A company’s operational capacity is determined by various factors, including its size, personnel composition, operational location, scheduling, transportation capacity, and financial and credit status, as governed by corresponding mechanisms [39]. These factors significantly impact the company’s ability to deliver services and maintain operational excellence. Smaller-scale enterprises may face challenges in meeting the demands of extensive public transportation services and may lack the resilience to manage risks effectively. The personnel composition is a critical factor, encompassing staff education levels, gender ratios, and age distribution. For transportation modes prioritizing public safety, higher educational standards for operators are crucial. The physical demands of certain roles may result in a lower proportion of female staff in transportation sectors, while age composition is essential for maintaining a balanced workforce. The operational location of transportation enterprises should prioritize public accessibility and convenience. Transportation scheduling must align with public needs, particularly during peak travel times, where adequate transportation capacity is critical for managing demand. Moreover, the financial and credit standing of operating enterprises is a key indicator of their operational capability and trustworthiness to traffic management departments. A robust financial position and favorable credit rating enhance the enterprise’s credibility and reliability, ensuring adherence to service expectations and regulatory requirements in the transportation sector.

Sudden events, such as extreme weather and natural disasters, can substantially disrupt public transportation and compromise safety. To address these challenges, operational management departments must adopt a proactive approach. Timely predictions and contingency planning are critical to mitigating the potential impact of such events on transportation services [40]. Operating enterprises must maintain a state of heightened crisis management awareness and capability. This preparedness enables enterprises to significantly reduce the likelihood and severity of traffic safety incidents during operational disruptions. Effective preparedness encompasses implementing robust response protocols, conducting ongoing training, and conducting regular drills to ensure personnel are equipped to manage emergencies. A proactive stance by both management departments and operating enterprises is crucial to protecting public transportation from the unpredictability of sudden events. Through strategic planning, vigilance, and preparedness, the transportation sector can maintain reliable and safe services despite adverse conditions.

Safety is paramount in all transportation modes. For vehicles such as buses and trains, service life longevity is a concern, but the potential for safety accidents poses an even greater risk [41]. Various factors contribute to this risk, including high operating speeds and prolonged operational periods. The dynamic nature of transportation increases the likelihood of safety incidents due to extended vehicle operation. Therefore, prioritizing safety measures is crucial to prevent accidents and safeguard passenger and public well-being. To mitigate these risks, regular maintenance and inspections of transportation vehicles are essential to ensure optimal condition. Implementing stringent safety protocols, providing drivers with training on high-speed situations, and educating passengers on emergency procedures can significantly reduce accident risk. Effective safety management necessitates collaboration between operators and regulatory bodies. In summary, while transportation vehicles facilitate movement over various distances, ensuring safety remains the primary responsibility of both operators and regulatory agencies, necessitating proactive measures to minimize risks and guarantee the well-being of passengers and the public.

Considering the analysis of risk factors affecting public transportation operations, a comprehensive approach to personnel training has been developed. This encompasses operational protocols, standardized task procedures, and specific on-the-job performance requirements. Robust supervision and management strategies, coupled with a clear rewards and penalties framework, ensure accountability and excellence. This study regards personnel training as a key factor influencing overall public transportation safety and efficiency. An inherent overlap between quality and safety management is acknowledged. For analytical clarity and consistency, however, quality management is treated as a distinct influencing factor throughout.

The primary risk factors influencing public transportation operations are summarized in Table 1, providing a concise foundation for further examination.

**Table 1:** Main influencing risk factors for public transportation operation.

Overall goal	Corresponding perspective	Influencing factor
Public transportation operation risk	Complete infrastructure	Reasonable planning
		Considerable funds
		Technology innovation
		Necessary testing
		Official approval
	Standardized process for personnel	Personnel training
		Operational skills
		Safety awareness
	Operational management	Rules and regulations
		Transport policy
		Supervision management
	Operational process requirements	Enterprise size
		Personnel composition
		Operating location and schedule
		Transportation capacity
		Financial and credit condition
	Risk response	Response mechanism
		Operational quality management mechanism

## 4. Entropy weighted DEMATEL analysis procedure

To enhance the analysis of the risk factors influencing public transportation operations as presented in Table 1, particular attention is given to the degree of influence and the underlying causal relationships of the identified factors. This paper leverages the strengths of entropy weight analysis for determining weights and the Decision Making Trial and Evaluation Laboratory (DEMATEL) method for elucidating internal causalities [25–30]. Consequently, we propose an integrated approach, termed the ‘entropy weighted DEMATEL analysis procedure,’ which combines the precision of entropy weight analysis with the relational insights of DEMATEL analysis. The procedural steps of this analysis are as follows:

**Procedure 1:** Influencing degree acquired on the basis of the entropy weight analysis process.

Utilizing the risk influencing factors compiled in Table 1, the subsequent analysis process aligns with the evaluation criteria presented in Table 2. Specifically, the number of evaluation criteria in Table 2 corresponds directly to the number of influencing factors listed in Table 1. Consequently, the weight analysis for the evaluation criteria in Table 2 is detailed as follows.

**Table 2:** Measurements for evaluated indicators of public transportation operation.

Evaluated indicator	Meaning	Measurement
Reasonable planning	Reasonable planning refers to the need for a good plan for the construction of public facilities, in order to ensure that the construction effect of public facilities is better. Generally speaking, the more reasonable the planning, the better the construction of public facilities.	For the convenience of measurement, use values ranging from 50 to 70 to measure reasonable planning. The larger the value, the more reasonable the planning.
Considerable funds	Considerable funds means that the construction of public facilities must be supported by sufficient funds. The larger the amount of funds, the stronger the construction of public facilities.	For the convenience of measurement, use values ranging from 20 to 35 to measure funds provided. The larger the value, the more considerable funds provided.
Technology innovation	Technology innovation refers to the degree of technological innovation used in the construction of public facilities, the higher the level of innovation, the better the infrastructure constructed,	Use percentage value to measure technology innovation. The larger the percentage value, the technology is more innovative.
Necessary testing	Necessary testing means that before public facilities that put into use, a certain amount of testing is undertaken to ensure the safety of corresponding utilization. The larger the measurement, the greater the safety performance of the facility.	Use percentage value to measure testing performance. The larger the percentage value, the better the testing performance.
Official approval	Official approval refers to support from relevant departments. The greater the support from government departments, the faster the construction speed of corresponding facilities.	Use value ranging from 70 to 90 to measure official approval. The larger the value obtained, the higher the approval from government departments.
Personnel training	Personnel training means that the training of corresponding public transportation operators. The higher the frequency of personnel training, the more beneficial for operation.	Use percentage value to measure personnel training. The larger the percentage value, the better the personnel training.
Operational skills	Operational skills means that essential skills for operational personnel. The better the operational skills, the better the public transportation operation	Use value from 20 to 30 to measure operational skills. The larger the value, the better the operational skills.
Safety awareness	Safety awareness the safety awareness of corresponding personnel during the operation process. The stronger the safety awareness, the more beneficial for public transportation operation.	Use percentage value to measure safety awareness. The larger the percentage value, the better the safety awareness.
Rules and regulations	Rules and regulations refer to the rules and regulations formulated by relevant departments for public transportation operations that need to be followed. The more standardized the rules and regulations, the more favorable it is for public transportation operation.	Use percentage value to measure rules and regulations. The larger the percentage value, the better the Rules and regulations made.
Transport policy	Transport policy refers to a series of policies and systems formulated by the national level for public transportation operation. The more comprehensive the policies and systems formulated by the government, the better the operation of public transportation is.	Use percentage value to measure the comprehension of rules and regulations made. The larger the percentage value, the better the for the rules and regulations made.
Supervision management	Supervision management means that supervision and management of transportation operations by relevant operational departments. The stronger the supervision and management work, the more conducive for traffic operation and management work.	Use percentage value to measure supervision management frequency. The larger the percentage value, the higher frequency supervised by the corresponding department.
Enterprise size	Enterprise size refers to the size of the enterprise and other related content to the scale of the enterprise. The larger the scale of the enterprise, the wider the scope of public transportation operation it provides.	Use value from 40 to 50 to measure enterprise size. The larger the value, the larger for the enterprise size.
Personnel composition	Personnel composition means that the proportion of men and women, gender ratio, age structure, and educational level in the composition of enterprise personnel. The younger the personnel composition of the enterprise, the higher the educational level, and the better the efficiency for public transportation operation.	Use value from 60 to 70 to measure personnel composition like age structure, and educational level. The larger the value, the better for personnel composition.
Operating location and schedule	Operating location and schedule refer to the location and timing of the services provided by the operating enterprise. The more convenient the location of the operating enterprise and the running time that can meet most requirements, it is easier to obtain public satisfaction.	Use value from 40 to 60 to measure 0perating location and schedule. The larger the value, the better for operating location and schedule.
Transportation capacity	Transportation capacity reflects the operational capacity of the operating enterprise. The larger the enterprise’s transportation capacity, the stronger for its operational capacity.	Use percentage value to measure transportation capacity. The larger the percentage value, the better for the transportation capacity.
Financial and credit condition	Financial and credit condition reflects the overall financial capability of the enterprise. The better financial and credit condition for the enterprise, the corresponding financial risk of the enterprise is also smaller.	Use value from 30 to 40 to measure financial and credit condition. The larger the value obtained, the better for the operational enterprise.
Response mechanism	Response mechanism refers to the speed at which relevant departments and enterprises respond to risks and the degree to which contingency plans are developed. Generally speaking, the better the corresponding mechanism, the more conducive it is to the safe operation of public transportation.	Use value from 60 to 70 to measure response mechanism. The larger the value obtained, the better for public transportation operation.
Operational quality management mechanism	Operational quality management mechanism means that comprehensive regulatory measures adopted by relevant departments. Comprehensive regulatory measures adopted better, the better for the operational quality management mechanism.	Use value from 55 to 65 to measure operational quality management mechanism. The larger the value obtained, the better for public transportation operation.

**Step 1:** Original evaluation for evaluated indicator.

Taking into account the multitude of evaluated factors, this study engages the expertise of five distinguished decision-makers to participate in the assessment of operational risk factors impacting public transportation. Their insights are crucial in providing a comprehensive and informed evaluation. Meanwhile, we assume that the expert i gives the original evaluation for the corresponding evaluated risk indicator j is $a_{ij}$, and i=1,2,⋯,5, j=1,2,⋯,18, all original risk evaluation $a_{ij}$ compose the original evaluated matrix A, and $A={\left(a_{ij}\right)}_{5\times 18}$.

**Step 2:** Standardization for the original evaluation.

Based on the original evaluation $a_{ij}$ (i=1,2,⋯,5, j=1,2,⋯,18) acquired in Step 1, with the help of calculation (1), we can acquire standardization result $a_{ij}^{\prime }$ (i=1,2,⋯,5, j=1,2,⋯,18) and the corresponding standardized matrix$A^{\prime }={\left(a_{ij}^{\prime }\right)}_{5\times 18}$.


$$a_{ij}^{\prime }=\frac{a_{ij}-\underset{i}{\min}\;a_{ij}}{\underset{i}{{\mathrm{maxa}}_{ij}}\;-\underset{i}{{\mathrm{mina}}_{ij}}\;}$$ (1)

In above formula (1), $\underset{i}{{\mathrm{maxa}}_{ij}}\;$ and $\underset{i}{mina_{ij}}\;$ represent the maximum and minimum evaluated values of expert i (i=1,2,⋯,5) for the analyzed risk factor j (j=1,2,⋯,18) respectively.

**Step 3:** Weight value computed on the account of Step 2.

Based on the standardization result $a_{ij}^{\prime }$ (i=1,2,⋯,5, j=1,2,⋯,18) shown in Step 2 for risk factor j, given the variance in the scales of various evaluation metrics, it is essential to undertake a normalization process for the outcomes of the standardized analysis, ensuring they fall within the range of 0–1. The pertinent computation formula is provided for reference. Moreover, with the computing result $p_{ij}$ (i=1,2,⋯,5, j=1,2,⋯,18) shown in formula (2), we can obtain the final weight value $\omega_{j}$ for corresponding risk factor j (j=1,2,⋯,18) from formula (3). Then, the influencing degree for the risk factor is acquired.


$$p_{ij}=\frac{a_{ij}^{\prime }}{\sum_{i=1}^{5}a_{ij}^{\prime }},\text{ j=1,2,}\cdots \text{,18}$$ (2)

$$w_{j}=\frac{1-(-\frac{1}{\ln18}\sum_{i=1}^{5}p_{ij}\cdot \ln p_{ij})}{18-\sum_{j=1}^{18}(-\frac{1}{\ln18}\sum_{i=1}^{5}p_{ij}\cdot \ln p_{ij})}(j=1,2,\ldots ,18)$$ (3)

**Procedure 2:** Internal causal relations of risk influencing factors on the account of DEMATEL analysis process [20–25].

DEMATEL is a powerful technique for dissecting the causal relationships among factors, primarily by assessing the relative influence each factor exerts on others. Utilizing the results derived from the entropy weight analysis, we can identify and filter out risk factors with minimal impact. Subsequently, the remaining influential factors, or evaluation indicators, are examined in terms of their mutual influence. By following a structured set of steps, we can elucidate the causal interconnections among the remaining evaluated risk factor.

**Step 1:** To get the direct influencing matrix.

In order to better analyze the impact relationship between influencing factors, generally speaking, we can use five numerical values of 0,1,2,3,4 (based on the content from DEMATEL method) to represent the d influence of the influencing factork (k=1,2,⋯,m) on the influencing factorl (l=1,2,⋯,n) (m and n represent the amount of the analyzed factors that put into contrast). To represent varying degrees of influence, the corresponding levels are categorized in ascending order of impact: from ‘no impact’ to ‘some impact’, progressing to ‘moderate impact’, then ‘significant impact’, and culminating in ‘high impact’. The corresponding degree of influence degree can be represented by the symbol$z_{kl}$ (k=1,2,⋯,m,l=1,2,⋯,n).The direct impact matrix formed by the influencing degree between two factors isZ, and $Z={\left(z_{kl}\right)}_{m\cdot n}$.

**Step 2:** Standardize operations for directly influencing matrix.

Based on Step 1, the normalization operation can perform by formula (4) to obtain normalization matrix$G={\left(g_{kl}\right)}_{m\cdot n}$.


$$g_{hl}=\frac{z_{hl}}{\underset{1\leq k\leq m}{\max}\;\sum_{l=1}^{n}z_{kl}}$$ (4)

**Step 3:** To calculate the comprehensive impact matrix.

On the basis of Step 2, the comprehensive impact matrix $T={\left(t_{kl}\right)}_{m\cdot n}$ obtained on formula (5).


$$T=G\cdot {\left(I-G\right)}^{-1}$$ (5)

In formula (5), the symbol I represents unit matrix.

**Step 4:** To compute the influencing degree and influenced degree.

Based on the results of the comprehensive impact matrixI, we can compute the influencing degree $id_{k}$ and influenced degree$ifd_{k}$ by the aid of formula (6) and formula (7). Moreover, based on the influencing degree $id_{k}$ and influenced degree $ifd_{k}$, we can get centrality degree $cd_{k}$ and reasoning degree $rd_{k}$ with formula (8) and formula (9).


$$id_{k}=\sum_{l=1}^{n}t_{kl}\left(k=1,2,\cdots ,m\text{ l=1,2,}\cdots \text{,n}\right)$$ (6)

$$ifd_{k}=\sum_{k=1}^{m}t_{kl}\left(k=1,2,\cdots ,m\text{ l=1,2,}\cdots \text{,n}\right)$$ (7)

$$cd_{k}=id_{k}+ifd_{k}\left(k=1,2,\cdots ,m\text{ l=1,2,}\cdots \text{,n}\right)$$ (8)

$$rd_{k}=id_{k}-ifd_{k}\left(k=1,2,\cdots ,m\text{ l=1,2,}\cdots \text{,n}\right)$$ (9)

**Fig 1:**
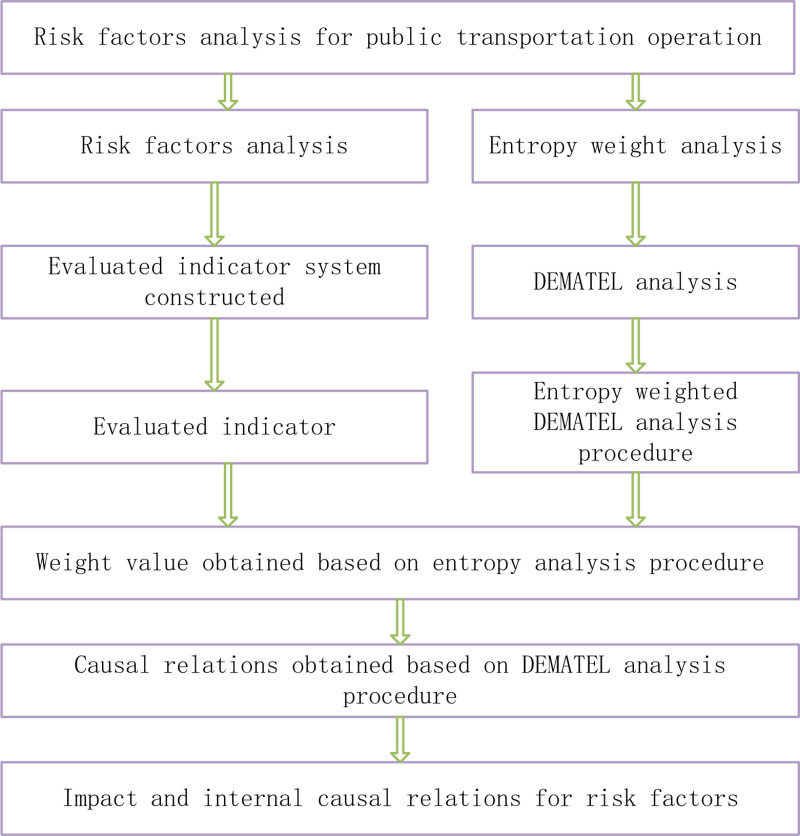
Entropy weighted DEMATEL analysis procedure for risk factors affect public transportation.

In summary, the entropy weight-DEMATEL decision-making process proposed in this paper is shown in Fig 1.

In Fig 1, the weight of the evaluated indicator is analyzed and the causal relationships among the evaluation indicators will also be analyzed.

## 5. Evaluation for operational risk factors

### 5.1 Measurement for relevant indicators

Owing to space constraints, the evaluation index system presented here aligns with the influencing factors detailed in Table 1. The dimensions of the evaluation are defined from the perspective of the influencing factors, with the corresponding factors serving as indicators within the evaluation system. Consequently, this paper does not reiterate the construction of the evaluation index system. Furthermore, providing detailed explanations of the meaning and measurement methods for each evaluated indicator is essential. These explanations serve as a reference for the five experts involved in the entropy analysis process, who provide initial evaluations for the different indicators. The relevant information is displayed in Table 2 (meaning is given to describe detailed information regarding the operational corresponding factor of the public transportation process, and the measurement is relevant to the original data given by the corresponding evaluated expert that participates in the subsequent entropy analysis process).

### 5.2 Evaluation for corresponding risk factors based on interval fuzzy information

In this paper, we invite five experts (relevant expert is randomly selected, including public operation decision-makers, public operation investors, public transportation practitioners, etc) to participate in the evaluation process to give corresponding original evaluation information Table 3 (Followed evaluated information given by five expert is based on measurement from Table 2 for corresponding evaluated indicator in Table 1, meanwhile, considering the uncertainty of decision-making, followed evaluated information is shown in interval information).

**Table 3:** Original interval fuzzy information.

Evaluated indicator	Expert 1	Expert 2	Expert 3	Expert 4	Expert 5
Reasonable planning	[53,62]	[56,67]	[59,68]	[54.64]	[52,61]
Considerable funds	[23,31]	[20,30]	[24,34]	[22.31]	[20,29]
Technology innovation	[78%,85%]	[69%,77%]	[71%,86%]	[67%,82%]	[75%,89%]
Necessary testing	[63%,72%]	[60%,71%]	[65%,81%]	[67%,83%]	[72%,81%]
Official approval	[75,85]	[71,87]	[73,84]	[75,89]	[73,88]
Personnel training	[67%,78%]	[68%,80%]	[73%,86%]	[75%,86%]	[72%,82%]
Operational skills	[23,27]	[21,30]	[22,29]	[20,27]	[23,29]
Safety awareness	[45%,57%]	[46%,58%]	[48%,61%]	[52%,72%]	[54%,73%]
Rules and regulations	[68%,81%]	[65%,82%]	[60%,79%]	[69%,83%]	[71%,84%]
Transport policy	[76%,83%]	[71%,80%]	[72%,85%]	[70%,84%]	[75%,86%]
Supervision management	[72%,83%]	[69%,76%]	[66%,75%]	[69%,77%]	[70%,83%]
Enterprise size	[43,48]	[40,46]	[41,49]	[43,59]	[44.50]
Personnel composition	[60,68]	[61,67]	[62,69]	[60,66]	[62,70]
Operating location and schedule	[42,56]	[40,52]	[45,57]	[44,59]	[46,59]
Transportation capacity	[63%,71%]	[62%,75%]	[62%,79%]	[65%,81%]	[61%,76%]
Financial and credit condition	[32,37]	[31,38]	[32,39]	[30.38]	[34,40]
Response mechanism	[61,69]	[62,67]	[62,68]	[61,69]	[65,70]
Operational quality management mechanism	[56,64]	[55,63]	[56,63]	[55,62]	[56,65]

In an effort to enhance the analysis of expert input, this paper employs the median of the fuzzy interval information presented in Table 3 to distill the most representative expert insights. The derived median expert information is then displayed in Table 4. Concurrently, the percentage data in Table 3 is converted into a percentage format for consistency and clarity.

**Table 4:** Median value for the fuzzy interval information from relevant experts.

Evaluated indicator	Expert 1	Expert 2	Expert 3	Expert 4	Expert 5
Reasonable planning	57.5	61.5	63.5	59	56.5
Considerable funds	27	25	29	26.5	24.5
Technology innovation	0.815	0.73	0.785	0.745	0.82
Necessary testing	0.675	0.655	0.73	0.75	0.765
Official approval	80	79	78.5	82	80.5
Personnel training	0.725	0,74	0,795	0,805	0.77
Operational skills	25	25.5	25.5	23.5	26
Safety awareness	0.51	0.52	0.545	0.62	0.635
Rules and regulations	0.745	0.735	0.695	0,76	0,775
Transport policy	0.795	0.755	0.785	0.77	0.805
Supervision management	0.775	0.725	0.705	0.73	0.765
Enterprise size	45.5	43	45	51	47
Personnel composition	64	64	65.5	63	66
Operating location and schedule	49	46	51	51.5	52.5
Transportation capacity	0.67	0.685	0.705	0.715	0.685
Financial and credit condition	34.5	34.5	35.5	34	37
Response mechanism	65	64.5	65	65	67.5
Operational quality management mechanism	60	59	59.5	58.5	60.5

Employing the entropy weight analysis process, we have determined the weight values of the evaluation indicators from the data in Table 4. On one hand, utilizing the formula (1), we have derived a standardized matrix for the risk factors under analysis.


$$P=\left(\begin{array}{ccc} & \begin{array}{ccccccccccccccccccccccccccccccccccccccccc} {}*35r0.1930 & 0.2045 & 0.2092 & 0.1888 & 0.2000 & 0.1890 & 0.1992 & 0.1802 & 0.2008\backslash 0.2064 & 0.1894 & 0.1874 & 0.1832 & 0.1975 & 0.1930 & 0.2032 & 0.1837 & 0.1981\backslash 0.2131 & 0.2197 & 0.2015 & 0.2042 & 0.1963 & 0.2073 & 0.2032 & 0.1926 & 0.1873\backslash 0.1980 & 0.2008 & 0.1913 & 0.2098 & 0.2050 & 0.2099 & 0.1873 & 0.2191 & 0.2049\backslash 0.1896 & 0.1856 & 0.2105 & 0.2140 & 0.2013 & 0.2008 & 0.2072 & 0.2244 & 0.2089\backslash \end{array},\backslash & \begin{array}{ccccccccccccccccccccccccccccccccccccccccc} {}*35r0.2033 & 0.2095 & 0.1965 & 0.1984 & 0.1960 & 0.1936 & 0.1966 & 0.1988 & 0.2017\backslash 0.1931 & 0.1959 & 0.1857 & 0.1984 & 0.1840 & 0.1980 & 0.1966 & 0.1972 & 0.1983\backslash 0.2008 & 0.1905 & 0.1944 & 0.2031 & 0.2040 & 0.2038 & 0.2023 & 0.1988 & 0.2000\backslash 0.1969 & 0.1973 & 0.2203 & 0.1953 & 0.2060 & 0.2066 & 0.1937 & 0.1988 & 0.1966\backslash 0.2059 & 0.2068 & 0.2030 & 0.2047 & 0.2100 & 0.1980 & 0.2108 & 0.2064 & 0.2034\backslash \end{array}\backslash \end{array}\right)$$

For above matrix P, due to space limitations, we only give objective weight value of the corresponding evaluated risk factor:


$$\omega_{j}=\left(\begin{array}{ccc} & 0.0560\;0.1088\;0.0653\;0.1083\;0.0070\;0.0484\;0.0357\;0.2490\;0.0405,\backslash & 0.0156\;0.0373\;0.0992\;0.0087\;0.0645\;0.0161\;0.0276\;0.0080\;0.0042\backslash \end{array}\right)$$

Utilizing the weight information derived from the entropy weight analysis for the aforementioned evaluation indicators, it becomes evident that the impact of ‘official approval’, ‘personnel composition’, ‘transportation capacity’, ‘response mechanism’, and ‘operational quality management mechanism’ is relatively minor compared to the other 13 evaluated indicators. ‘Official approval’ is an essential infrastructure measure for public transportation. ‘Personnel composition’ is a vital element of enterprise scale. Likewise, ‘transportation policy’, ‘response mechanism’, and ‘operational quality management mechanism’ are integral to the operational process of public transportation, while ‘transportation capacity’ is a key aspect of transportation requirements, and the ‘operational quality management mechanism’ is part of the supervisory management framework. Therefore, considering the impact of these evaluated indicators, when examining the causal relationships among the remaining indicators, we can reasonably disregard the influence of ‘official approval’, ‘personnel composition’, ‘transportation capacity’, ‘response mechanism’, and ‘operational quality management mechanism’ on the interval causal relationships.

Furthermore, leveraging the weight analysis facilitated by the entropy weight method, we are poised to delve deeper into the interval causal relationships among the evaluated indicators, employing the structured approach of the DEMATEL analysis procedure.

Following the analysis steps of the DEMATEL procedure outlined earlier, we can initially construct the direct influence matrix.


$$Z=\left(\begin{array}{ccccccccccccccccccccccccccccccccccccccccccccccccccccccccccccccccccccccccccccccccccccccccccccccccccccccccccccccccccccccccccccccccccccccccccccccccccccccccccccc} 0 & 4 & 3 & 2 & 2 & 1 & 2 & 2 & 2 & 2 & 3 & 3 & 2\backslash 3 & 0 & 3 & 3 & 2 & 1 & 1 & 2 & 2 & 3 & 3 & 2 & 4\backslash 2 & 2 & 0 & 2 & 1 & 1 & 1 & 1 & 2 & 1 & 3 & 1 & 2\backslash 2 & 3 & 3 & 0 & 2 & 3 & 2 & 2 & 2 & 1 & 2 & 3 & 2\backslash 2 & 3 & 2 & 2 & 0 & 3 & 2 & 2 & 2 & 1 & 2 & 2 & 2\backslash 2 & 2 & 3 & 3 & 4 & 0 & 2 & 1 & 2 & 2 & 3 & 2 & 1\backslash 3 & 2 & 3 & 2 & 1 & 2 & 0 & 3 & 2 & 1 & 2 & 3 & 2\backslash 1 & 2 & 2 & 3 & 1 & 2 & 2 & 0 & 3 & 1 & 2 & 1 & 2\backslash 2 & 3 & 3 & 2 & 1 & 3 & 2 & 3 & 0 & 2 & 4 & 3 & 2\backslash 4 & 2 & 3 & 3 & 2 & 3 & 3 & 4 & 2 & 0 & 2 & 3 & 2\backslash 3 & 3 & 4 & 2 & 3 & 2 & 2 & 2 & 3 & 4 & 0 & 3 & 3\backslash 2 & 3 & 2 & 3 & 2 & 3 & 2 & 2 & 1 & 3 & 3 & 0 & 4\backslash 2 & 3 & 4 & 3 & 2 & 3 & 2 & 2 & 1 & 2 & 3 & 4 & 0\backslash \end{array}\right)$$

Subsequently, we proceed to calculate the normalized results, ensuring that the data is adjusted to a common scale for effective comparison and analysis.


$$G=\left(\begin{array}{ccccccccccccccccccccccccccccccccccccccccccccccccccccccccccccccccccccccccccccccccccccccccccccccccccccccccccccccccccccccccccccccccccccccccccccccccccccccccccccc} {}*35r0 & 0.1176 & 0.0882 & 0.0588 & 0.0588 & 0.0294 & 0.0588 & 0.0588 & 0.0588 & 0.0588 & 0.0882 & 0.0882 & 0.0588\backslash 0.0882 & 0 & 0.0882 & 0.0882 & 0.0588 & 0.0294 & 0.0294 & 0.0588 & 0.0588 & 0.0882 & 0.0882 & 0.0588 & 0.1176\backslash 0.0588 & 0.0588 & 0 & 0.0588 & 0.0294 & 0.0294 & 0.0294 & 0.0294 & 0.0588 & 0.0294 & 0.0882 & 0.0294 & 0.0588\backslash 0.0588 & 0.0882 & 0.0882 & 0 & 0.0588 & 0.0882 & 0.0588 & 0.0588 & 0.0588 & 0.0294 & 0.0588 & 0.0882 & 0.0588\backslash 0.0588 & 0.0882 & 0.0588 & 0.0588 & 0 & 0.0882 & 0.0588 & 0.0588 & 0.0588 & 0.0294 & 0.0588 & 0.0588 & 0.0588\backslash 0.0588 & 0.0588 & 0.0882 & 0.0882 & 0.1176 & 0 & 0.0588 & 0.0294 & 0.0588 & 0.0588 & 0.0882 & 0.0588 & 0.0294\backslash 0.0882 & 0.0588 & 0.0882 & 0.0588 & 0.0294 & 0.0588 & 0 & 0.0882 & 0.0588 & 0.0294 & 0.0588 & 0.0882 & 0.0588\backslash 0.0294 & 0.0588 & 0.0588 & 0.0882 & 0.0294 & 0.0588 & 0.0588 & 0 & 0.0882 & 0.0294 & 0.0588 & 0.0294 & 0.0588\backslash 0.0588 & 0.0882 & 0.0882 & 0.0588 & 0.0294 & 0.0882 & 0.0588 & 0.0882 & 0 & 0.0588 & 0.1176 & 0.0882 & 0.0588\backslash 0.1176 & 0.0588 & 0.0882 & 0.0882 & 0.0588 & 0.0882 & 0.0882 & 0.1176 & 0.0588 & 0 & 0.0588 & 0.0882 & 0.0588\backslash 0.0882 & 0.0882 & 0.1176 & 0.0588 & 0.0882 & 0.0588 & 0.0588 & 0.0588 & 0.0882 & 0.1176 & 0 & 0.0882 & 0.0882\backslash 0.0588 & 0.0882 & 0.0588 & 0.0882 & 0.0588 & 0.0882 & 0.0588 & 0.0588 & 0.0294 & 0.0882 & 0.0882 & 0 & 0.1176\backslash 0.0588 & 0.0882 & 0.1176 & 0.0882 & 0.0588 & 0.0882 & 0.0588 & 0.0588 & 0.0294 & 0.0588 & 0.0882 & 0.1176 & 0\backslash \end{array}\right)$$

Hence, we can obtain the comprehensive influencing matrix:


$$T=\left(\begin{array}{cccccccccccccc} & \text{0}.2876 0\text{.4337 0}.4370 0\text{.3618 0}.2944 0\text{.2993 0}.2880 0\text{.3165 0}.3035 0\text{.3021 0}.4093 0\text{.3844 0}.3523\backslash & \text{0}.3797 0\text{.3409 0}.4530 0\text{.3997 0}.3047 0\text{.3126 0}.2731 0\text{.3273 0}.3126 0\text{.3358 0}.4218 0\text{.3738 0}.4119\backslash & \text{0}.2575 0\text{.2865 0}.2526 0\text{.2697 0}.1985 0\text{.2193 0}.1930 0\text{.2122 0}.2306 0\text{.2032 0}.3129 0.2447 0.2622\backslash & \text{0}.3271 0\text{.3919 0}.4195 0\text{.2921 0}.2854 0\text{.3392 0}.2769 0.3014 0.2912 0.2622 0.3689 0.3690 0.3352\backslash & \text{0}.3098 0.3720 0.3725 0.3287 0.2155 0.3227 0.2629 0.2861 0.2766 0.2470 0.3482 0.3253 0.3162\backslash & \text{0}.3301 0.3682 0.4206 0.3720 0.3386 0.2604 0.2790 0.2782 0.2935 0.2877 0.3935 0.3450 0.3085\backslash & \text{0}.3416 0.3549 0.4064 0.3361 0.2480 0.3024 0.2133 0.3189 0.2828 0.2526 0.3567 0.3581 0.3241\backslash & \text{0}.2547 0.3129 0.3380 0.3231 0.2184 0.2710 0.2399 0.2061 0.2787 0.2210 0.3154 0.2702 0.2855\backslash & \text{0}.3612 0.4284 0.4616 0.3829 0.2873 0.3698 0.3046 0.3587 0.2661 0.3191 0.4572 0.4040 0.3691\backslash & \text{0}.4328 0.4288 0.4864 0.4317 0.3294 0.3909 0.3497 0.4053 0.3402 0.2761 0.4295 0.4270 0.3882\backslash & \text{0}.4257 0.4718 0.5321 0.4221 0.3677 0.3808 0.3359 0.3693 0.3773 0.3992 0.3927 0.4448 0.4317\backslash & \text{0}.3651 0.4321 0.4405 0.4127 0.3172 0.3750 0.3081 0.3360 0.2954 0.3453 0.4325 0.3290 0.4221\backslash & \text{0}.3673 0.4359 0.4936 0.4155 0.3190 0.3763 0.3090 0.3365 0.2986 0.3224 0.4377 0.4359 0.3205\backslash \end{array}\right)$$

Next, utilizing the comprehensive impact matrix derived from the previous steps, we can determine the influencing degree and the influenced degree for each factor.


$$id_{k}=\left(\text{4}.4402 5\text{.0580 5}.5138 4\text{.7481 3}.7241 4\text{.2197 3}.6334 4\text{.0525 3}.8471 3\text{.7737 5}.0763 4\text{.7112 4}.5275\right)$$

$$ifd_{k}=\left(\begin{array}{cccccccccccccc} & \text{4}.4699\backslash & \text{4}.6469\backslash & \text{3}.1429\backslash & \text{4}.2600\backslash & \text{3}.9835\backslash & \text{4}.2753\backslash & \text{4}.0959\backslash & \text{3}.5349\backslash & \text{4}.7700\backslash & \text{5}.1160\backslash & \text{5}.3511\backslash & \text{4}.8110\backslash & \text{4}.8682\backslash \end{array}\right)$$

Ultimately, we derive the centrality degree and reasoning degree, which are pivotal for understanding the relative significance and causal influence of each factor within the system.


$$cd_{k}=\left(\begin{array}{ccc} & 8.9101\;9.7049\;8.6567\;9.0081\;7.7076\;8.495\;7.7293\;7.5874\;\backslash & 8.6171\;8.8897\;10.4274\;9.5222\;9.3957\backslash \end{array}\right)$$

$$rd_{k}=\left(\begin{array}{ccc} & -0.0297\;0.4111\;2.3709\;0.4881\;-0.2594\;-0.0556\;-0.4625\;0.5176\;\backslash & -0.9229\;-1.3423\;-0.2748\;-0.0998\;-0.3407\backslash \end{array}\right)$$

Based on the calculated centrality and causality degrees of the evaluated indicators, it is evident that ‘enterprise scale’ exerts the most substantial impact, with ‘considerable funds’, ‘operating location’, and ‘schedule’ also ranking highly compared to the other ten indicators. Given the general operations of public transportation, ‘enterprise scale’ emerges as the most significant risk factor influencing the operational process. On the other hand, according to the positive and negative outcomes of the cause degree for the evaluation indices, ‘considerable funds’, ‘technology innovation’, ‘necessary testing’, and ‘rules and regulation’ are identified as causal factors that impact public transportation operations. Conversely, ‘reasonable planning’, ‘personnel training’, ‘operational skills’, ‘safety awareness’, ‘transportation policy’, ‘supervision management’, ‘enterprise scale’, ‘operating location and schedule’, and ‘financial and credit condition’ are categorized as outcome factors that influence the operation of public transportation.Therefore, from a risk management perspective in public transportation operations, it is imperative to mitigate the causal factors of risk to effectively control the resulting outcome factors triggered by associated risks.

## 6. Discussion, conclusions and managerial implications

The risk factors for public transport are part of a dynamic and constantly changing process. Through analysis of the influencing factors that affect the operational process of public transportation, with the help of entropy-weighted DEMATEL method analysis, we find that safety awareness is the most significant factor, highlighting the importance of enhancing the safety consciousness of all individuals involved in the operation of public transport, which should receive attention from the whole society to reduce unnecessary traffic safety hazards. In terms of combined decision-making in this paper, we try to involve more experts (for example, more than 10 or even more scholars), and more comprehensive influencing factors are interesting research that can be done in the future.

The significance of safety awareness as the primary factor is acknowledged, but equal attention should be given to other influencing factors involved in public transportation operations. A heightened sense of safety, driven by human initiative, can substantially mitigate unforeseen accident risks. Financial resources, ranked second, play a crucial role in advancing infrastructure development and maintaining safety. Adequate funding is essential for ongoing construction projects and safe operation of public transportation systems. Necessary testing, ranked third, underscores the importance of safety performance assessments for transportation infrastructure prior to operation. The DEMATEL analysis elucidates internal causal relationships among evaluated factors, identifying considerable funds, technological innovation, necessary testing, and rules and regulations as causal risk factors influencing public transportation operations. Conversely, reasonable planning, personnel training, operational skills, safety awareness, transportation policy, supervision management, enterprise scale, operating location and schedule, and financial and credit conditions are categorized as outcome risk factors. This analysis highlights the multifaceted nature of risk in public transportation and provides a structured approach to addressing these risks, enhancing overall safety and efficiency. Effective management of key factors such as safety awareness, financial resources, and necessary testing can potentially reduce uncertainty impact on public transport operations. Tailored measures must be implemented to mitigate risks associated with other influencing factors. From an operational perspective, controlling causal risk factors (substantial funding, technological innovation, rigorous testing, and adherence to regulations) is imperative for shaping the operational process and ensuring smooth functioning. Addressing outcome risk factors (strategic planning, workforce training, operational proficiency, safety consciousness, policy frameworks, oversight mechanisms, organizational scale, operational scheduling, and financial health) is essential to reduce their impact. A thorough analysis of risk factor interrelationships and weight values enables prioritization and effective management of public transportation risks. This strategic approach enhances operational efficiency and bolsters resilience against unforeseen challenges.

In public transportation safety operations, humans are identified as the most critical active factor using the entropy weight DEMATEL method. Controlling operational risk awareness is crucial for creating a safer environment through increased publicity (Risk awareness is the primary principle in addressing public transportation safety and is also an important aspect that public transportation operating departments need to resolve), financial investment in risk management (In the actual decision-making process, it is essential to consider the cultivation and guidance of risk awareness and actively enhance safety risk awareness. Continuously increasing investment in public transportation operations is the key to building a strong transportation nation and ensuring the safe development of transportation operations), and comprehensive application of related systems and regulatory measures (Only with more comprehensive traffic regulations and systems can the safe operation of transportation be institutionally guaranteed).
